# Dapagliflozin’s impact on hormonal regulation and ketogenesis in type 1 diabetes: a randomised controlled crossover trial

**DOI:** 10.1007/s00125-025-06481-9

**Published:** 2025-07-09

**Authors:** Andreas Gübeli, Nicole Steiner, Andreas Limacher, Déborah Mathis, Andreas Melmer, Markus Laimer

**Affiliations:** 1 Department of Diabetes, Endocrinology, Clinical Nutrition & Metabolism, Inselspital, Bern University Hospital, University of Bern, Bern, Switzerland; 2 Department of Clinical Research, University of Bern, Bern, Switzerland; 3 Swiss Paraplegic Research, Nottwil, Switzerland; 4 University Institute of Clinical Chemistry, Inselspital, Bern University Hospital, University of Bern, Bern, Switzerland

**Keywords:** Dapagliflozin, Diabetic ketoacidosis, GLP-1, Glucagon, Ketogenesis, Somatostatin, Type 1 diabetes

## Abstract

**Aims/hypothesis:**

This study aimed to assess the impact of adding dapagliflozin to insulin therapy on key hormonal determinants of glucose regulation and ketogenesis. We hypothesise that dapagliflozin increases glucagon-like peptide 1 (GLP-1), glucagon and ketone body concentrations, based on the results of a pilot study.

**Methods:**

The study was designed as a randomised, placebo-controlled, open-label, crossover intervention study with two periods (dapagliflozin and placebo intake), including patients of the Department of Diabetes, Endocrinology, Clinical Nutrition & Metabolism, Inselspital, Bern University Hospital, University of Bern. Individuals with type 1 diabetes (C-peptide concentrations <0.1 nmol/l) with a duration >5 years and a BMI of 20–29 kg/m^2^ were included. They received 10 mg of dapagliflozin or placebo daily for 7 days throughout two independent treatment periods, separated by a 14 day washout period. Allocation was done by a computed randomisation tool (REDCap), without blinding of the participants or the investigators. On day 7 of each treatment period, hyperinsulinaemic–euglycaemic clamps (HECs) and OGTT clamps (OGTTCs) were performed to assess changes in the secretion of GLP-1, glucagon, somatostatin and total ketone bodies. The objective was to evaluate the effects of adding the sodium–glucose cotransporter 2 (SGLT2) inhibitor dapagliflozin to insulin therapy on GLP-1 during OGTTC (primary endpoint), GLP-1 secretion during HEC, and glucagon, somatostatin and ketogenesis during OGTTC and HEC (secondary endpoints). The primary endpoint was concentrations of GLP-1 during OGTTC. Secondary endpoints included GLP-1 during HEC and glucagon, somatostatin and ketone body concentrations during OGTTC and HEC.

**Results:**

A total of 13 individuals with type 1 diabetes were included and randomised. All of them received dapagliflozin and placebo, finished the sequences per protocol and were analysed per protocol. GLP-1 concentrations did not differ significantly between treatments in the OGTTC (median [IQR] dapagliflozin 192.8 [129.8–257.2] pmol/l vs placebo 176.3 [138.4–227.4] pmol/l; *p*=0.7) or HEC (median [IQR] dapagliflozin 208.6 [133.6–294.0] pmol/l vs placebo 203.1 [150.2–291.8] pmol/l; *p*=0.7). Glucagon concentrations did not significantly differ between treatments in the OGTTC (median [IQR] dapagliflozin 1.54 [0.84–3.68] ng/l vs placebo 1.54 [0.82–4.64] ng/l; *p*=0.8) or HEC (median [IQR] dapagliflozin 1.59 [0.87–3.54] ng/l vs placebo 1.63 [0.91–3.96] ng/l; *p*=0.3). Somatostatin concentrations remained comparable between treatments during the HEC (median [IQR] dapagliflozin 41.1 [26.8–73.8] pmol/l vs placebo 47.0 [23.0–77.6] pmol/l; *p*=0.2) and OGTTC (median [IQR] dapagliflozin 51.1 [31.1–77.0] pmol/l vs placebo 45.3 [30.0–70.5] pmol/l; *p*=0.2). Plasma ketone bodies were higher with dapagliflozin during the HEC (median [IQR] dapagliflozin 0.15 [0.04–0.47] mmol/l vs placebo 0.03 [0.01–0.12] mmol/l; *p*<0.001) and OGTTC (median [IQR] dapagliflozin 0.10 [0.03–0.22] mmol/l vs placebo 0.03 [0.01–0.12] mmol/l; *p*<0.001).

**Conclusions/interpretation:**

Short-term dapagliflozin treatment in type 1 diabetes increases plasma ketone concentrations without affecting the secretion of GLP-1, glucagon or somatostatin. Higher ketone body concentrations highlight the elevated risk of diabetic ketoacidosis associated with the adjunct intake of dapagliflozin.

**Trial registration:**

ClinicalTrials.gov NCT04035031.

**Funding:**

Swiss National Science Foundation, project number 32003B_185019.

**Graphical Abstract:**

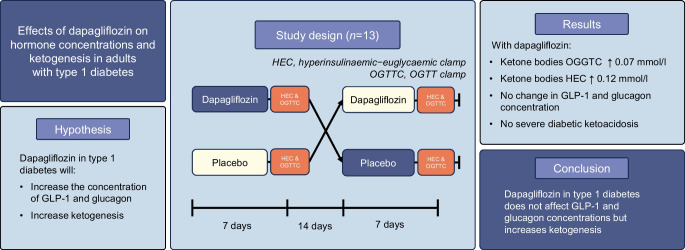

**Supplementary Information:**

The online version contains peer-reviewed but unedited supplementary material available at 10.1007/s00125-025-06481-9.


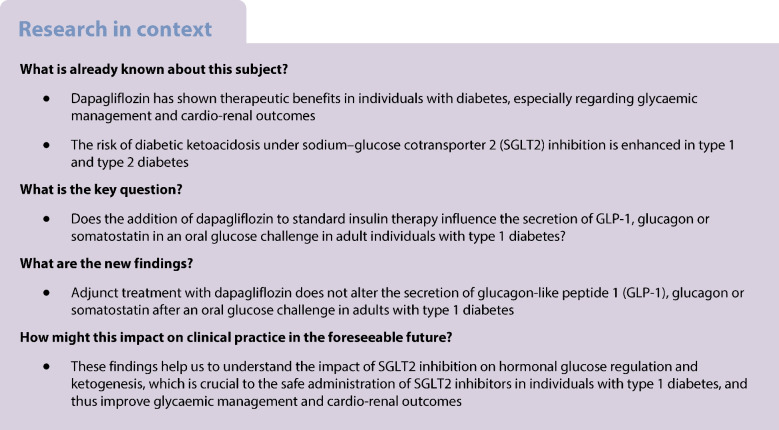


## Introduction

### Background

Adequate insulin dosing in type 1 diabetes poses significant challenges: achieving near-normal glycaemic management needs to be balanced with minimising the risk of acute hypoglycaemia and long-term complications. Sodium–glucose cotransporter 2 (SGLT2) inhibitors have emerged as potential adjuncts to insulin therapy in type 1 diabetes due to their insulin-independent increase of renal glucose excretion.

Dapagliflozin, a highly selective SGLT2 inhibitor, has demonstrated therapeutic benefits in individuals with type 1 diabetes by reducing postprandial glucose concentrations, albuminuria and blood pressure, along with promoting weight loss [1–3]. These effects are primarily attributed to its induction of glycosuria and improved insulin sensitivity. However, the use of SGLT2 inhibitors in type 1 diabetes is associated with an increased risk of diabetic ketoacidosis, a serious and potentially life-threatening complication [2, 3].

At the hormonal level, glucagon-like peptide 1 (GLP-1) and glucagon in particular play a key role in glucose homeostasis alongside insulin. GLP-1, an incretin hormone secreted by intestinal L cells in response to nutrient ingestion, was found to increase insulin sensitivity and to suppress glucagon release [4, 5]. Glucagon, as produced by pancreatic alpha cells, is crucial for maintaining glucose homeostasis by stimulating hepatic glucose production through glycogenolysis and gluconeogenesis. In type 1 diabetes, dysregulated glucagon secretion may exacerbate hyperglycaemia and contribute to metabolic excursions [6]. Modulation of these hormones may therefore influence glycaemic management in individuals with type 1 diabetes.

### Objectives

This study was performed in adults with type 1 diabetes to evaluate the effects of dapagliflozin on hormonal determinants of glucose homeostasis and ketogenesis when added to standard insulin treatment. Hyperinsulinaemic–euglycaemic clamps (HECs) and OGTT clamps (OGTTCs) were conducted to compare the impact of dapagliflozin on circulating concentrations of GLP-1 (primary endpoint) and on glucagon, somatostatin and total ketone bodies (secondary endpoints) with placebo treatment. Understanding these effects may provide new insights into potential benefits and risks associated with dapagliflozin in type 1 diabetes.

## Methods

### Trial design

**Fig. 1:**
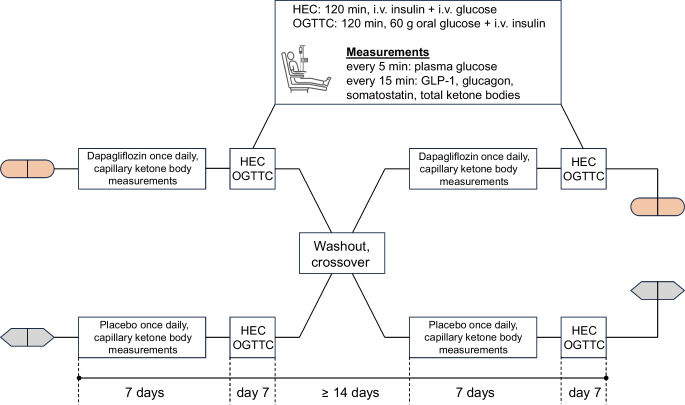
Study design

The trial was designed as an open-label, crossover study (Fig. 1). A total of 13 individuals with type 1 diabetes were randomised to receive either 10 mg of dapagliflozin or placebo as an adjunct to insulin therapy for an initial 7 day period, followed by the alternative treatment for another 7 days. A 14 day washout period was implemented between the two intervention periods to avoid carry-over effects.

### Sample size

A priori sample size calculation was based on the primary outcome: the median GLP-1 concentration during the OGTTC. A former pilot study performed by our research group showed an SD of 6.32 pmol/l for GLP-1 during OGTTC [7]. A difference of 10 pmol/l between the two treatment phases was deemed clinically relevant, and a conservative SD of 10 pmol/l was assumed. Based on a paired-means test, we calculated a required number of 13 individuals to detect this difference at a two-sided alpha level of 0.05 with a power of 90%. This sample size calculation was conservative, as repeated measures of GLP-1 over 120 min may provide statistical power higher than 90%.

### Participants

**Fig. 2:**
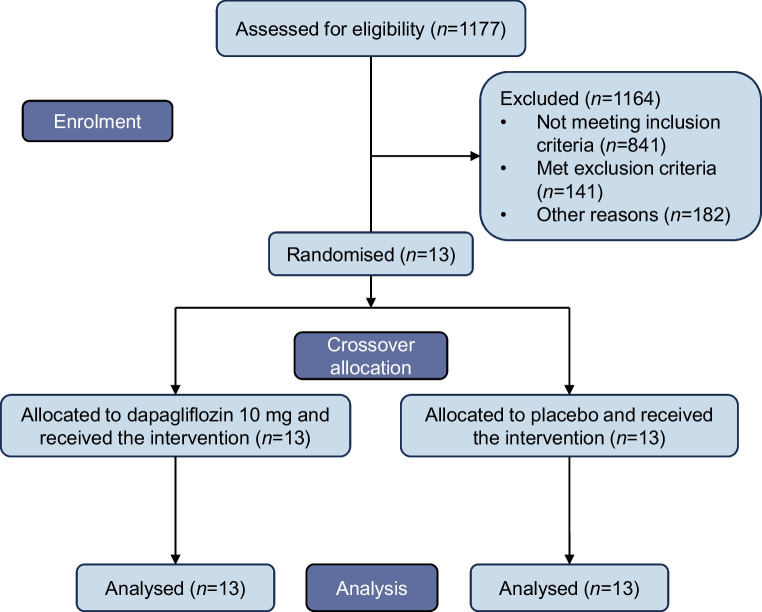
CONSORT flow diagram of study recruitment and randomisation. Following recruitment, 13 individuals with type 1 diabetes were randomised to receive either 10 mg of dapagliflozin or placebo as an adjunct to insulin therapy for an initial 7 day period, followed by the alternative treatment for another 7 days

A total of 1177 outpatients of the Department of Diabetes, Endocrinology, Clinical Nutrition & Metabolism, Inselspital, Bern University Hospital, University of Bern, were assessed for eligibility. In total, 982 candidates were excluded based on the inclusion or exclusion criteria. The inclusion criteria were a duration of type 1 diabetes >5 years, a BMI of 20–29 kg/m^2^ and C-peptide concentrations <0.1 nmol/l. The exclusion criteria were a diagnosis of renal or hepatic dysfunction, a history of any type of malignancy, the intake of drugs influencing glucose homeostasis during the last 3 months, alcohol or drug abuse, active smoking (five or more cigarettes per day) and pregnancy. Thirteen individuals gave consent after detailed explanation of the trial (Fig. 2). The study sample was broadly representative of the general Swiss population in terms of sex, gender, ethnicity and socioeconomic status. However, the limited sample size constrained the ability to demonstrate age representativeness. Aside from gender, these demographic characteristics were not systematically assessed. Gender was determined via self-report and was neither used as an inclusion or exclusion criterion nor explicitly considered in the trial design or recruitment process. The study was approved by the Cantonal Ethics Committee Bern prior to conducting any study-related actions involving potentially eligible participants. All procedures performed involving human participants were in accordance with the ethical standards of the institutional and/or national research committees and with the 1964 Declaration of Helsinki and its later amendments.

### Randomisation

Randomisation in a 1:1 ratio was conducted electronically via REDCap, an electronic database that was distributed and maintained by the clinical trial unit of the University of Bern. As the study was unblinded, both investigators and participants were informed of the scheduled intervention sequence.

### Examinations: HEC and OGTTC

During both intervention periods, participants were asked to ensure that their macronutrient intake was similar in both treatment periods and to abstain from alcohol. On day 7 of each intervention period, a HEC was performed, followed by an OGTTC. Blood sampling was conducted after an 8 h overnight fasting period. In brief, during the HEC, insulin was infused at a rate of 40 mU/m^2^ body surface/min for 120 min. The body surface was calculated using the formula of DuBois et al [8]:

$${{\text{Body surface }\left[{\text{m}}^{2}\right]=0.007184\times \text{height }[\text{cm}]}^{0.725}\times \text{weight }[\text{kg}]}^{0.425}$$

Blood glucose concentrations were measured every 5 min and titrated at 7.0 mmol/l (±2.0 mmol/l as the predefined target range) by intravenous infusion of 20% glucose. After completion of the HEC, blood glucose concentrations were maintained within the target range for at least 60 min. Subsequently, participants ingested 50 g of oral d-glucose dissolved in 300 ml of tap water for the OGTTC (time point 0). Blood glucose concentrations were measured every 5 min. Insulin requirements for 50 g of d-glucose were calculated as follows:

$$\left(1\right)\, \text{Carbohydrate factor}=\frac{500}{\text{total daily insulin dose }[\text{U}]}$$

$$(2)\, \text{Insulin required }[\text{U}]=\frac{\text{glucose load }[\text{g}]}{\text{carbohydrate factor}}$$

The calculated total insulin dose was administered via three decreasing infusion rates over a total of 60 min (50% from 0 to 20 min, followed by 30% from 21 to 40 min and 20% from 41 to 60 min of the OGTTC). Blood sampling for primary and secondary endpoints was performed every 15 min during 120 min of the HEC and 120 min of the OGTTC.

### Outcomes

#### Primary outcome

The primary outcome was the median differences in GLP-1 concentrations observed during the OGTTC after the intake of dapagliflozin and placebo. GLP-1 was measured every 15 min during 120 min of the OGTTC.

#### Secondary outcomes

The secondary outcomes were the median differences in GLP-1 concentrations measured every 15 min during 120 min of the HEC, the median differences in glucagon and somatostatin concentrations measured every 15 min during 120 min of the HEC and OGTTC, and the median concentrations of total ketone body measured at baseline and every 15 min during 120 min of the HEC and OGTTC after dapagliflozin vs placebo intake.

#### Exploratory outcome

The exploratory outcome was the plasma glucose concentrations during the OGTTC measured after dapagliflozin vs placebo intake.

### Changes from protocol

In the original study protocol, the AUC for GLP-1 was defined as the primary outcome. However, to facilitate the interpretation of results, we decided to report unit-based outcomes of a repeated-measures mixed-model analysis, leveraging all time points instead of the AUC. AUC analysis is a way of aggregating results, while a repeated-measures mixed-model analysis provides greater statistical robustness together with increased readability. AUC values are provided in the electronic supplementary material (ESM Table 1).

### Biochemical analysis

For pre-analytic conservation of GLP-1 and glucagon, a P800 blood collection tube holding a proprietary mixture of protease inhibitors (BD Biosciences, San Jose, CA, USA) was used. For measurement of total ketone bodies and somatostatin, EDTA-coated plasma samples were collected. Active (7-36) GLP-1 concentrations were determined using a human GLP-1 ELISA kit (cat. no. EHGLP; Thermo Fisher Scientific, Basel, Switzerland) with a detection range of 47.4–6066.0 pmol/l. Glucagon concentrations were measured using a human glucagon ELISA kit (cat. no. EHGCG; Thermo Fisher Scientific, Basel, Switzerland) with a detection range of 2.03–130.00 ng/l. Somatostatin concentrations were measured using a human somatostatin ELISA kit (cat. no. abx153137; Abbexa, Cambridge, UK) with a detection range of 3.8–305.5 pmol/l. Concentrations of β-hydroxybutyrate and acetoacetate were determined via LC-MS at the central laboratory unit of Bern University Hospital.

### Statistical analysis

#### Primary analysis

The primary analysis was based on the full analysis set (FAS). We depicted the time course of the primary and secondary outcomes in line plots separately for the active and placebo treatments. We presented the median outcome concentrations with lower and upper quartiles separately per treatment. Mean differences in log-transformed primary and secondary outcome concentrations were calculated using a mixed-effects repeated-measures linear model. The model included fixed effects for the treatment (dapagliflozin vs placebo), treatment period (first vs second) and time points (categorical), as well as a random intercept for participants. We used restricted maximum likelihood (REML) and the Satterthwaite method for calculating 95% CIs and *p* values. We used an autoregressive correlation structure determined as best-fitting by the Akaike’s information criterion (AIC). We back-transformed the model coefficients and confidence limits to display a geometric mean ratio with a 95% CI between the two treatments that can be interpreted on the original scale.

#### Secondary analyses

A secondary analysis was performed based on the per-protocol patient set (ESM Table 2). We presented the median AUC and quartiles of the primary and secondary outcomes separated per treatment period. The AUC was calculated using the linear trapezoidal method. We analysed differences in the log-transformed AUC using a mixed-effects linear model based on the FAS. The model included fixed effects for the treatment (dapagliflozin vs placebo) and treatment period (first vs second), as well as a random intercept for participants. We used REML and the Satterthwaite method for calculating 95% CIs and *p* values. We used an unstructured correlation matrix that fitted the data best as assessed by the AIC. We back-transformed the model coefficients and confidence limits to display a geometric mean ratio with a 95% CI between the two treatments that can be interpreted on the original scale.

#### Additional analyses

In order to exclude a carry-over effect and/or treatment-by-period interaction, we tested the primary outcome model for an interaction between period and intervention; no evidence of such an interaction was found. Additionally, we calculated the within-participant intraclass correlation (ESM Table 3) of primary and secondary outcomes from mixed-effects repeated-measures linear models. Normality of model residuals was assessed using Q–Q plots. Due to the log-normal distribution of the outcome measures, data were log-transformed before analysis.

#### Post hoc exploratory analysis

OGTTC plasma glucose concentrations were added as exploratory outcomes post hoc and were analysed like the primary outcome, except that no log-transformation was necessary.

## Results

A total of 13 (six male and seven female) participants were enrolled between January 2020 and September 2020. All participants completed the study and were analysed per protocol for the prespecified outcomes (Fig. 1). Following recruitment and the clinical testing, the trial was closed as planned. Participants’ demographic and baseline data are summarised in Table 1.

**Table 1:** Demographic data of study participants

Characteristic	Overall *N*=13	Placebo first *N*=6	Dapagliflozin first *N*=7
Age, years	38.2 ± 11.4	39.2 ± 14.4	37.3 ± 9.3
Gender, *n* (%)			
Male	7 (54)	4 (67)	3 (43)
Female	6 (46)	2 (33)	4 (57)
BMI, kg/m^2^	23.8 ± 2.2	23.7 ± 2.7	23.8 ± 2.1
Time since type 1 diabetes diagnosis, years	23.2 ± 10.8	19.3 ± 13.1	26.6 ± 8.0
Use of continuous glucose monitoring device, *n* (%)	13 (100)	6 (100)	7 (100)
Total daily basal insulin dose, U	19.4 ± 6.7	17.1 ± 7.2	21.3 ± 6.1
Total daily bolus insulin dose, U	26.3 ± 12.3	32.1 ± 14.6	21.3 ± 8.0
Total daily insulin dose, U	45.6 ± 16.4	49.2 ± 20.9	42.6 ± 12.4
Type of insulin used, *n* (%)			
Novorapid	5 (38)	2 (33)	3 (43)
Fiasp	7 (54)	3 (50)	4 (57)
Humalog	1 (8)	1 (17)	0 (0)
Others	0 (0)	0 (0)	0 (0)
Plasma glucose, mmol/l	9.1 ± 2.3	8.0 ± 1.1	10.0 ± 2.7
HbA_1c_, mmol/mol	57.4 ± 15.4	49.7 ± 7.7	62.8 ± 18.5
HbA_1c_, %	7.4 ± 1.4	6.7 ± 0.7	7.9 ± 1.7
C-peptide ≤0.1 nmol/l, yes, *n* (%)	13 (100)	6 (100)	7 (100)
TSH, mU/l	2.1 ± 1.5	2.6 ± 2.1	1.7 ± 0.6
GLP-1, pmol/l	278.4 ± 199.6	285.6 ± 306.3	273.2 ± 102.0
(Missing)	1	1	0
Glucagon, ng/l	2.79 ± 2.69	3.18 ± 3.24	2.47 ± 2.40
(Missing)	2	1	1
Somatostatin, pmol/l	55.1 ± 35.9	40.7 ± 37.5	67.5 ± 32.0

Data are presented as mean ± SD or *n* (%)

TSH, thyroid-stimulating hormone

GLP-1 concentrations did not differ significantly between treatment periods during the OGTTC (median [IQR] dapagliflozin 192.8 [129.8–257.2] pmol/l vs placebo 176.3 [138.4–227.4] pmol/l; *p*=0.7) or HEC (median [IQR] dapagliflozin 208.6 [133.6–294.0] pmol/l vs placebo 203.1 [150.2–291.8] pmol/l; *p*=0.7) (Table 2; presented as a line plot in ESM Fig. 1).

**Table 2:** Concentrations of primary and secondary endpoints during the OGTTC and HEC interventions

Outcome	Placebo^a^	Dapaglifozin^a^	Ratio^b^ (95% CI)	*p* value
OGTTC GLP-1 (pmol/l)	176.3 (138.4–227.4)	192.8 (129.8–257.2)	0.97 (0.86–1.10)	0.7
HEC GLP-1 (pmol/l)	203.1 (150.2–291.8)	208.6 (133.6–294.0)	0.98 (0.88–1.09)	0.7
OGTTC glucagon (ng/l)	1.54 (0.82–4.64)	1.54 (0.84–3.68)	0.98 (0.82–1.17)	0.8
HEC glucagon (ng/l)	1.63 (0.91–3.96)	1.59 (0.87–3.54)	0.92 (0.79–1.08)	0.3
OGTTC somatostatin (pmol/l)	45.3 (30.0–70.5)	51.1 (31.1–77.0)	1.13 (0.93–1.37)	0.2
HEC somatostatin (pmol/l)	47.0 (23.0–77.6)	41.1 (26.8–73.8)	1.17 (0.94–1.45)	0.2
OGTTC plasma ketone (mmol/l)	0.03 (0.01–0.12)	0.10 (0.03–0.22)	2.01 (1.41–2.87)	<0.001
HEC plasma ketone (mmol/l)	0.03 (0.01–0.12)	0.15 (0.04–0.47)	2.70 (1.90–3.84)	<0.001

^a^ Median (IQR), calculated through a repeated-measures mixed-model analysis

^b^ Geometric mean ratio (exponentiated model coefficient)

Glucagon concentrations were similar between treatment periods during the OGTTC (median [IQR] dapagliflozin 1.54 [0.84–3.68] ng/l vs placebo 1.54 [0.82–4.64] ng/l; *p*=0.8) and HEC (median [IQR] dapagliflozin 1.59 [0.87–3.54] ng/l vs placebo 1.63 [0.91–3.96] ng/l; *p*=0.3). (Table 2, ESM Fig. 1).

Somatostatin concentrations remained statistically comparable between the dapagliflozin and placebo groups during the HEC (median [IQR] dapagliflozin 41.1 [26.8–73.8] pmol/l vs placebo 47.0 [23.0–77.6] pmol/l; *p*=0.2) and OGTTC (median [IQR] dapagliflozin 51.1 [31.1–77.0] pmol/l vs placebo 45.3 [30.0–70.5] pmol/l; *p*=0.2) (Table 2, ESM Fig. 1).

During the OGTTC, concentrations of plasma ketone bodies were significantly higher after the intake of dapagliflozin than after placebo (median [IQR] dapagliflozin 0.10 [0.03–0.22] mmol/l vs placebo 0.03 [0.01–0.12] mmol/l; *p*<0.001). Concentrations of plasma ketone bodies measured during the HEC were significantly higher after dapagliflozin treatment than after placebo (median [IQR] dapagliflozin 0.15 [0.04–0.47] mmol/l vs placebo 0.03 [0.01–0.12] mmol/l; *p*<0.001) (Table 2; ESM Fig. 1).

As an exploratory outcome, plasma glucose concentrations measured during the OGTTC were significantly lower after the intake of dapagliflozin than after placebo (median [IQR] dapagliflozin 7.84 [6.08–9.40] mmol/l vs placebo 8.53 [6.94–10.54] mmol/l; *p*<0.001) (ESM Table 4).

One uncomplicated urinary tract infection and several higher ketone body concentration measurements were observed during both the unsupervised intake period and the day the HEC and OGTTC were conducted. All ketone-related adverse events (elevation of capillary or plasma total ketone body concentrations above 0.5 mmol/l) were classified as mild and asymptomatic. No severe adverse events occurred during the study period.

## Discussion

Short-term administration of dapagliflozin as an adjunct to standard insulin therapy had no effect on the secretion of GLP-1, glucagon or somatostatin. However, significantly higher concentrations of ketone bodies were observed than with placebo.

This result contrasts with a review by Takebayashi and Inukai, which suggests that SGLT2 inhibitors may increase GLP-1 secretion in type 2 diabetes, and with the incidental findings of our previous pilot study [7, 9]. This discrepancy in GLP-1 secretion from individuals with type 2 diabetes could be attributed to the absence of functioning beta cells in type 1 diabetes, affecting the enteroinsular axis. These findings suggest that the effects of dapagliflozin on glucose homeostasis and ketogenesis are independent of incretin modulation. Furthermore, GLP-1 secretion appears to be blunted depending on the degree of insulin resistance, as was found in various studies comparing individuals with normal glucose tolerance, impaired glucose tolerance and type 2 diabetes, although to a heterogeneous degree [4, 10, 11].

Glucagon concentrations were similar after dapagliflozin and placebo intake throughout the OGTTC and HEC. This finding contradicts several studies suggesting elevated glucagon secretion associated with SGLT2 inhibition [6, 12, 13]. Our hypothesis is that the increased renal glucose excretion after the intake of dapagliflozin will lower basal insulin requirements. In individuals with type 1 diabetes, in whom virtually no functional beta-cell population remains and insulin is administered exogenously, reduced exogenous insulin dosing associated with dapagliflozin could have led to higher glucagon concentrations through reduced suppression of alpha-cell activity and a counter-regulatory response [14]. We could not confirm that theory in vivo. One possible explanation is that the difference in insulin requirements may be too small to influence glucagon secretion. Alternatively, it may occur only in a hypoglycaemic state, which we did not examine.

Furthermore, Bonner et al have demonstrated SGLT2 expression in pancreatic alpha cells, leading to enhanced cellular glucagon secretion, when inhibited [12]. However, we did not observe an effect of those mechanisms in vivo. This discrepancy may be attributed to lower local dapagliflozin concentrations in vivo than in vitro or to the fact that the effects of the enhanced glucagon secretion were too small to produce detectable changes at a systemic level.

We confirmed significantly higher ketone body concentrations after dapagliflozin treatment, corresponding to the known increased risk of ketosis and diabetic ketoacidosis associated with SGLT2 inhibition [14].

Stimulation of ketogenesis appeared to be independent of changes in glucagon secretion, as previously noted. Several mechanisms may explain a glucagon-independent increase in ketogenesis with dapagliflozin treatment. Due to the glucose-lowering effect of dapagliflozin, exogenous insulin doses may be reduced, resulting in a diminished anabolic stimulus and an increase in counter-regulatory hormones. This may lead to an enhanced energy expenditure, which could stimulate lipolysis and ketogenesis. Another possible mechanism is that dapagliflozin reduced renal ketone body clearance by increasing tubular reabsorption [15].

However, despite the significant rise, plasma ketone body concentrations remained within moderate ranges, and no cases of diabetic ketoacidosis were observed, which is a crucial finding in terms of safety concerning SGLT2 inhibitors in type 1 diabetes. The modest increase in ketone bodies highlights the need for careful participant selection, education on ketone monitoring and adherence to mitigation strategies, as previously specified by Goldenberg et al [16].

The significantly reduced plasma glucose concentrations observed during the OGTTC after dapagliflozin intake suggest that insulin requirements were reduced during treatment with dapagliflozin. This aligns with previous studies demonstrating that SGLT2 inhibitors may decrease daily insulin requirements by enhancing urinary glucose excretion. This insulin-sparing effect is clinically relevant, as it may reduce the risk of hypoglycaemia and alleviate the burden of intensive insulin therapy in type 1 diabetes management [1, 2, 13].

The balance between the benefits of dapagliflozin, such as improved glycaemic management, and the risks associated with increased ketogenesis is a critical consideration for clinicians. They should be aware of these hormonal effects when prescribing dapagliflozin and implement appropriate monitoring protocols. Understanding the hormonal effects of dapagliflozin can aid in developing personalised treatment regimens that balance glycaemic benefits and risks.

Further research involving larger, more diverse, populations and longer follow-up periods is needed to better understand the long-term effects of dapagliflozin on glucose homeostasis and the risk of diabetic ketoacidosis in type 1 diabetes. As no detectable differences were expected due to the small sample size, gender was not included in the analyses. However, potential differences should be further investigated in future studies with larger populations. Investigations focusing on strategies for the safe implementation of SGLT2 inhibitors in type 1 diabetes, including personalised insulin dose adjustments and ketone monitoring protocols, are also warranted.

### Limitations

The present study has several limitations. The open-label study design may have influenced both the data collection and the behaviour of participants during the intake period of dapagliflozin or placebo. However, a Hawthorne effect is also observed during double-blinded designs. Moreover, as dapagliflozin has regularly been shown to increase ketogenesis in numerous studies, participants would have most likely unblinded themselves via the daily measurement of capillary ketone body concentrations, which was required for safety reasons. Second, GLP-1 concentrations are vastly modulated by macronutrient composition, food processing, body weight and insulin resistance [4, 11]. Although participants were instructed to maintain similar meals during both treatment periods, their macronutrient intake was not standardised. This may have increased inter-individual variability of GLP-1 secretion during the HEC and OGTTC, depending on the macronutrient composition of the preceding meals and individual duration of digestion and absorption.

The median blood glucose levels during the HEC were not strictly euglycaemic (median [IQR] dapagliflozin 6.70 [6.09–7.34] mmol/l vs placebo 6.75 [6.05–7.36] mmol/l; ESM Table 4). However, since glucose measurements were statistically identical between both treatment arms and as blood glucose concentrations only moderately deviated from euglycaemia, we trust in the robustness of the present results.

Another limitation is that plasma insulin concentrations were not measured during the OGTTC or HEC. Variations in plasma insulin concentrations may have influenced the secretion of GLP-1 and glucagon. However, since we confirmed that the participants had no endogenous insulin production (C-peptide <0.1 nmol/l) and as we primed all surfaces on infusion material to prevent insulin loss, we assume that the infused insulin doses strongly corresponded to the effective plasma insulin concentrations. Given that the infused insulin doses were standardised, a significant impact on the results seems unlikely.

### Conclusion

In conclusion, the addition of dapagliflozin to standard insulin treatment in type 1 diabetes had no effect on GLP-1, glucagon or somatostatin, yet stimulated ketone body production. While the reduction of plasma glucose observed after dapagliflozin treatment may indicate improved glycaemic management, it also contributes to an enhanced risk of diabetic ketoacidosis. These findings provide insights into the effects of dapagliflozin in a selected population of adult individuals with type 1 diabetes. Even though higher concentrations of ketone bodies were found, diabetic ketoacidosis did not occur in this trial. However, clinicians should carefully weigh the benefits of improved glycaemic management against the potential risk of diabetic ketoacidosis, ensuring that patients are appropriately educated and monitored. Personalised approaches and adherence to safety protocols are essential when considering SGLT2 inhibitors as an adjunct therapy in type 1 diabetes management.

## Supplementary Information

Below is the link to the electronic supplementary material.

ESM (PDF 882 KB)

## Data Availability

Raw data are not publicly available to preserve participants’ privacy. They can be accessed in the form of a justified request via the directory of the Department of Diabetes, Endocrinology, Clinical Nutrition & Metabolism, Inselspital, Bern University Hospital, University of Bern, Switzerland (https://udem.insel.ch/).

## Abbreviations

AIC: Akaike’s information criterion
eCRF: Electronic case report form
FAS: Full analysis set
GLP-1: Glucagon-like peptide 1
HEC: Hyperinsulinaemic–euglycaemic clamp
OGTTC: OGTT clamp
REML: Restricted maximum likelihood
SGLT2: Sodium–glucose cotransporter 2
