# Supplementary material for: Dapagliflozin’s impact on hormonal regulation and ketogenesis in type 1 diabetes: a randomised controlled crossover trial
Source: Diabetologia. 2025 Jul 9;68(10):2116–25. doi: 10.1007/s00125-025-06481-9 (PMC12423202; doi:10.1007/s00125-025-06481-9)

Dapagliflozin's impact on hormonal regulation and ketogenesis in type 1 diabetes: a randomised controlled crossover trial

Andreas Gübeli[1], Nicole Steiner[1], Andreas Limacher[2,3], Déborah Mathis[4], Andreas Melmer[1], Markus Laimer[1]

1. Department of Diabetes, Endocrinology, Clinical Nutrition & Metabolism, Inselspital, Bern University Hospital, University of Bern, Switzerland
2. Department of Clinical Research, University of Bern, Switzerland
3. Swiss Paraplegic Research, Nottwil, Switzerland
4. University Institute of Clinical Chemistry, Inselspital, Bern University Hospital, University of Bern, Switzerland

Andreas Gübeli and Nicole Steiner contributed equally to this work as first authors. Andreas Melmer and Markus Laimer contributed equally as senior authors.

Corresponding author

Markus Laimer

E-mail: Markus.Laimer@insel.ch

ORCID: A. Gübeli 0009-0006-2530-8388, A. Limacher 0000-0002-9094-9476, D. Mathis: 0000-0002-2326-7782, A. Melmer 0000-0001-8085-8768, M. Laimer 0000-0002-7622-0822

Electronic supplementary material

ESM Table 1: Analysis of area under the curves (AUC)

Outcome	Placebo[1]	Dapaglifozin[1]	Ratio[2] (95%-CI)	p-value
AUC OGTTC GLP-1 (pmol/l×min)	23668 (19099 - 26252)	24224 (18555 - 32349)	0.95 (0.82 - 1.10)	0.5
AUC HEC GLP-1 (pmol/l×min)	23213 (20346 - 35579)	25553 (17222 - 35265)	0.97 (0.79 - 1.20)	0.8
AUC OGTTC Glucagon (ng/l×min)	167 (85 - 493)	189 (110 - 439)	0.96 (0.69 - 1.33)	0.8
AUC HEC Glucagon (ng/l×min)	170 (93 - 470)	209 (97 - 417)	0.95 (0.74 - 1.23)	0.7
AUC OGTTC Somatostatin (pmol/l×min)	5270 (3711 - 9778)	6627 (4705 - 10496)	1.17 (0.83 - 1.64)	0.3
AUC HEC Somatostatin (pmol/l×min)	6940 (2828 - 8974)	5506 (3772 - 9318)	1.04 (0.76 - 1.43)	0.8
AUC OGTTC Plasma Ketone (mmol/l×min)	4.95 (3.15 - 9.90)	11.55 (6.45 - 22.65)	1.69 (0.88 - 3.27)	0.11
AUC HEC Plasma Ketone (mmol/l×min)	6.45 (2.25 - 17.25)	29.10 (11.40 - 65.85)	3.91 (2.35 - 6.50)	<0.001

[1] Median (ICR)

[2] Geometric mean ratio (exponentiated model coefficient)

ESM Table 2: Per-protocol (PP) analysis of primary and secondary outcomes

The secondary per-protocol analysis is based on the PP set (9 patients)

Outcome	Placebo[1]	Dapagliflozin[1]	Ratio[2] (95%-CI)	p-value
OGTTC GLP-1 (pmol/l)	192.4 (128.1 - 263.8)	193.2 (139.8 - 260.0)	0.93 (0.78 - 1.10)	0.4
HEC GLP-1 (pmol/l)	240.6 (144.4 - 328.3)	213.1 (145.6 - 309.7)	0.96 (0.84 - 1.08)	0.5
OGTTC Glucagon (ng/l)	2.05 (0.68 – 4.42)	1.52 (0.70 – 3.92)	1.08 (0.89 - 1.30)	0.4
HEC Glucagon (ng/l)	1.89 (0.77 – 3.80)	1.55 (0.74 – 4.47)	1.03 (0.88 - 1.22)	0.7
OGTTC Somatostatin (pmol/l)	40.3 (26.6 - 63.1)	51.8 (31.1 - 76.1)	1.02 (0.84 - 1.24)	0.8
HEC Somatostatin (pmol/l)	45.0 (21.6 - 76.5)	41.1 (27.7 - 81.2)	1.17 (0.94 - 1.46)	0.2
OGTTC Plasma Ketone (mmol/l)	0.05 (0.02 - 0.15)	0.11 (0.05 - 0.22)	1.98 (1.42 - 2.77)	<0.001
HEC Plasma Ketone (mmol/l)	0.06 (0.02 - 0.20)	0.19 (0.08 - 0.53)	2.88 (2.04 - 4.08)	<0.001

[1] Median (IQR)

[2] Geometric mean ratio (exponentiated model coefficient)

ESM Table 3: The within-patient interclass correlation (ICC)

Outcome	ICC
OGTTC GLP-1 (pmol/l)	0.74
HEC GLP-1 (pmol/l)	0.84
OGTTC Glucagon (ng/l)	0.87
HEC Glucagon (ng/l)	0.88
OGTTC Somatostatin (pmol/l)	0.55
HEC Somatostatin (pmol/l)	0.47
OGTTC Plasma Ketone (mmol/l)	0.70
HEC Plasma Ketone (mmol/l)	0.72

ESM Table 4: Glucose Level during HEC and OGTTC

Outcome	Placebo[1]	Dapaglifozin[1]	Difference (95%-CI)	p-value
OGTTC plasma glucose (mmol/l)	8.53 (6.94 - 10.54)	7.84 (6.08 - 9.40)	-1.08 (-1.48 - -0.68)	< 0.001
HEC plasma glucose (mmol/l)	6.75 (6.05 - 7.36)	6.70 (6.09 - 7.34)	0.01 (-0.16 - 0.17)	>0.9

[1] Median (IQR)

ESM Figure 1: Line plot of primary and secondary outcomes

Time course of primary and secondary outcomes. Each patient is shown and coloured individually

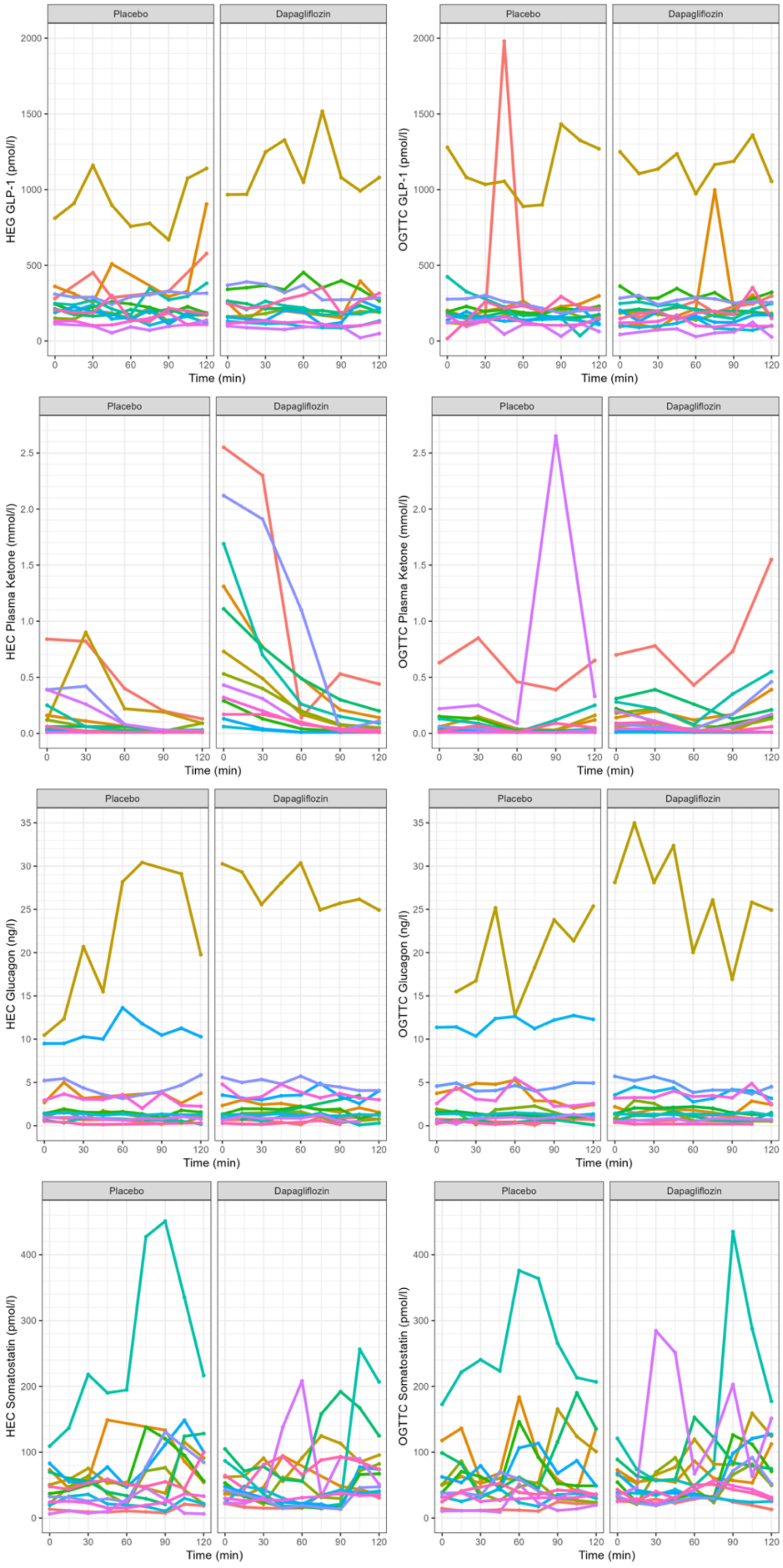

Supplement: Supplementary file 1 — ESM (PDF 882 KB) [file 125_2025_6481_MOESM1_ESM.pdf]
